# STAT3 signaling is required for anti-OX40/TGF-β receptor blockade-mediated regression of large established tumors

**DOI:** 10.1186/2051-1426-1-S1-P68

**Published:** 2013-11-07

**Authors:** Emmanuel T Akporiaye, Christopher Tucker, Todd Triplett, Kendra Garrison, Lihong Sun, Leona Ling, Andrew Weinberg

**Affiliations:** 1 Earle A. Chiles Research Institute, Providence Cancer Center, Portland, OR, USA; 2 Molecular Microbiology and Immunology, Oregon Health and Sciences University, Portland, OR, USA; 3 Oncology and Cell Signaling, Biogen Idec, Cambridge, MA, USA

## 

OX40 (CD134, TNFRSF4), a member of the tumor necrosis factor receptor (TNFR) superfamily is expressed on activated CD4+ and CD8+ T cells. In pre-clinical tumor models, agonist OX40 antibody (αOX40) therapy is often successful at treating small tumors but is less effective once the tumors have become established. For a tumor immunotherapy to be successful it will most likely require not only an agonist to boost effector T cell function but also an antagonist to eliminate T cell suppression. In this study, we show that agonist OX40 antibody synergizes with an orally bioavailable inhibitor of TGF-β (SM16) to elicit complete regression of large established tumors, resulting in long-term survival in 40-85% of αOX40/SM16 treated mice in two murine tumor models. Evaluation of tumor infiltrating T cells showed that SM16/αOX40 dual therapy resulted in an increase in OX40 and Granzyme B-expressing CD8+ T cells undergoing proliferation and which produced greater levels of IFNγ. We also found that this dual treatment led to an increase in pSTAT3 staining in both CD4+ and CD8+ T cells isolated from tumors. Therefore, we tested whether deletion of STAT3 in OX40 expressing cells would impact this potent combination therapy, since others have shown that pSTAT3 up-regulation is detrimental to T cell function within the tumor microenvironment. Surprisingly, deletion of STAT3 decreased the therapeutic efficacy of this combination therapy, suggesting that immune enhancement of T cells within tumor-bearing mice is reliant on signals through STAT3 to gain their full therapeutic potential.

